# The Intercultural Mediator as a Bridge in Healthcare Professional–Migrant Patient Care Relationships: A Qualitative Study

**DOI:** 10.3390/healthcare14131903

**Published:** 2026-06-30

**Authors:** Gabriele Caggianelli, Arianna Anzini, Irene Dello Iacono, Giovanni Cangelosi, Rita Patrizia Tomasin, Luigi Apuzzo, Marcello Torre, Alessandro Stievano, Dhurata Ivziku

**Affiliations:** 1 Department of Healthcare Professions, Azienda Ospedaliera Complesso Ospedaliero San Giovanni Addolorata, Via dell’Amba Aradam, 8, 00184 Rome, Italy; caggianelligabriele@gmail.com (G.C.); aanzini@hsangiovanni.roma.it (A.A.); rptomasin@hsangiovanni.roma.it (R.P.T.); 2 JBI Italy Evidence-Based Practice and Health Research Centre, 00136 Rome, Italy; alessandro.stievano@gmail.com; 3 School of Pharmacy, Experimental Medicine and “Stefania Scuri” Public Health Department, Via Madonna delle Carceri 9, 62032 Camerino, Italy; 4 Health Workforce Standards and Staffing Needs, Organizational Models of the Health Professions Unit, National Agency for Regional Health Services (AGENAS), Via Toscana 12, 00187 Rome, Italy; luigiapuzzo@hotmail.it; 5 Department of Healthcare Professions, ASST Sette-Laghi, 21100 Varese, Italy; marcello.torre@asst-settelaghi.it; 6 Center of Excellence for Nursing Culture and Research, Order of Nursing Professions of Rome, Viale degli Ammiragli, 67 sc.B, 00136 Rome, Italy; 7 Department of Healthcare Professions, Fondazione Policlinico Universitario Campus Bio-Medico, Via Álvaro del Portillo, 200, 00128 Rome, Italy; d.ivziku@policlinicocampus.it; 8 Faculty of Medical Sciences, AAB College, 10000 Pristina, Kosovo

**Keywords:** intercultural mediator, healthcare professionals, migrant patient, culturally competent care, qualitative research, health equity

## Abstract

**Highlights:**

**What are the main findings?**

- Intercultural mediators act as relational bridges, enhancing communication, trust, and culturally sensitive care in migrant patient interactions.
- Their contribution remains limited by organizational barriers, including poor integration into teams and inconsistent availability.

**What are the implications of the main findings?**

- Structurally integrating intercultural mediators into healthcare teams may improve equity, communication quality, and continuity of care.
- Intercultural training and clear role definitions are needed to optimize collaboration and support culturally competent care delivery.

**Abstract:**

**Background/Objectives**: This study aims to describe the experiences of healthcare professionals collaborating with Intercultural Mediators (IMs) in the care relationships with migrant patients. **Methods**: This study is a qualitative descriptive study. A total of 13 healthcare professionals (9 nurses and 4 physicians) working in two public hospitals in Italy participated in face-to-face semi-structured interviews between June and July 2024. Interviews were audio-recorded, transcribed verbatim and analyzed using inductive qualitative content analysis. **Results**: Three main categories emerged: (1) the experience of healthcare professionals in the relationship with migrant patients; (2) the linguistic and intercultural mediator as a supportive relational role; and (3) limitations in language and intercultural mediation. Participants described difficulties in mutual understanding due to cultural and linguistic barriers and emphasized the central role of the mediator as a bridge between diverse cultural worlds. IMs were described as essential in promoting trust, improving therapeutic adherence, and supporting culturally sensitive communication. However, organizational challenges included limited availability of IMs, poor integration into clinical teams, and the lack of structured, continuous support system. **Conclusions**: IMs are perceived as key actors in facilitating communication, trust, and therapeutic adherence with migrant patients. Yet their integration within healthcare teams remains underutilized due to organizational barriers. The findings inform managers and policymakers about the necessity of structurally integrating IMs within clinical teams and implementing intercultural competence programmes to ensure equitable and culturally sensitive care for migrant patients.

## 1. Introduction

Globalization and the intersections of socio-political, demographic, economic, and environmental forces have intensified migration flows from low- and middle-income countries to more industrialized countries and reshaped healthcare systems in destination countries [1]. Over the past few decades, international migrants have increased substantially [1,2], contributing to growing cultural and linguistic diversity and disparities in health outcomes within healthcare settings [3]. In Italy, migrants represent a significant proportion of the population and include heterogeneous communities with diverse health needs [4].

Although migration itself is not synonymous with vulnerability, migrant people may encounter barriers that hinder timely access to services and continuity of care, including language barriers, stigmatization, a lack of documentation, unfamiliarity with the healthcare system, and socio-economic vulnerability [5]. These barriers lead to delayed diagnoses, inadequate treatments, and avoidable hospitalizations [6,7] contributing to persistent inequalities in health status and healthcare quality [8].

Communication is a core component of safe, effective, and person-centred care and represents a fundamental dimension of the caring relationship, particularly in nursing practice [9]. However, in cross-cultural encounters, communication may become fragile. Healthcare professionals must navigate linguistic barriers while also interpreting culturally embedded meanings of health, illness and care practices to build effective therapeutic relationships [3,10]. When communication is compromised, it can be more difficult to establish therapeutic relationships and share the care plan, compromising continuity of care and health outcomes among migrant populations [11].

To address these challenges, several healthcare systems have introduced the role of the intercultural mediator (IM) to facilitate communication and support the therapeutic relationship between healthcare professionals and migrant patients [12]. An intercultural mediator is a trained professional who facilitates communication and mutual understanding by addressing both the linguistic and sociocultural dimensions of care [12].

Although the role of an IM is often confused with that of an interpreter [13], the two functions differ. The interpreter’s primary function is linguistic translation, whereas the IM encompasses a broader scope, including bridging cultural expectations and explanatory models, supporting conflict prevention and resolution, fostering trust, providing health education, counselling and advocacy and offering psychosocial support [14].

Existing evidence suggests that IMs contribute to improving communication and patient–healthcare professional relationships [15], reducing perceived discrimination [16], facilitating access to healthcare services, and supporting smoother healthcare pathways [17,18]. Intercultural mediation has also been associated with improved identification of gender-based violence compared to other professional roles, highlighting its potential relevance for safeguarding and equity-oriented care [19]. Despite these documented benefits, the integration of IMs within healthcare organizations remains heterogeneous. There is no internationally standardized professional profile and training pathways vary across contexts in duration and content, leading to differences in competencies and role expectations [14,20].

In Italy, although IMs are trained through regionally accredited vocational programmes and/or university degrees [21], the profession is not regulated by a single national framework, and practices differ across regions and employers [14,22]. Furthermore, the inclusion of IMs frequently depends on temporary funding or outsourcing arrangements, resulting in fragmented implementation and limited continuity within care teams [23,24]. Such organizational variability may shape how intercultural mediation is enacted in clinical practice and how healthcare professionals perceive its usefulness, boundaries and limitations.

While an increasing body of research has examined the functions and organizational impact of IMs [25], less attention has been devoted to understanding how collaboration with IMs is experienced by healthcare professionals in everyday clinical practice. In particular, little is known about how nurses and physicians perceive IMs’ contribution within structured hospital settings and how such collaboration shapes therapeutic relationships and care processes. Understanding these experiences is important because the way intercultural mediation is enacted in daily practice may influence communication processes, relational dynamics, and the organization of care for migrant patients. Exploring healthcare professionals’ perspectives may contribute to clarifying facilitators and challenges in collaboration, informing strategies for more consistent integration of IMs within healthcare teams, and ultimately supporting equitable and person-centred care in multicultural contexts.

The aim of this study was to describe the experiences of healthcare professionals collaborating with IMs in the care relationships with migrant patients.

## 2. Materials and Methods

### 2.1. Study Design

A qualitative descriptive study was conducted, in line with Sandelowski et al. (2000) [26], to describe healthcare professionals’ experiences of caring for migrant patients in collaboration with IMs. This approach enables a comprehensive, low-inference description of experiences as they are reported, using participants’ own language and remaining close to the surface of words and events. Qualitative description is particularly appropriate for examining the who, what, where, and why of phenomena occurring in everyday clinical practice [26]. In this study, it facilitated the systematic exploration of healthcare professionals’ perceptions, emotions, and professional reflections related to intercultural mediation in real-world care settings, generating findings directly relevant to clinical practice and organizational decision-making.

The research was reported in accordance with the Consolidated Criteria for Reporting Qualitative Research (COREQ) [27] (Supplementary File S1).

### 2.2. Participants

The study was conducted in two large public referral hospitals located in northern and central Italy, providing secondary and tertiary care services, with approximately 700 and 1200 inpatient beds, respectively. Both hospitals deliver a wide range of inpatient and outpatient services, including medical and surgical care, emergency services, rehabilitation, day-hospital and day-surgery pathways, pediatric contexts, and multidisciplinary outpatient care. These settings were selected because they are in urban areas characterized by significant migratory flows and provide routine access to structured intercultural mediation services integrated into everyday clinical practice. Approximately 15% of patients in these hospitals belong to culturally and linguistically diverse populations ensuring regular exposure to intercultural mediation during patient care.

A purposive sample of healthcare professionals was recruited to obtain information-rich cases relevant to the study aim. Inclusion criteria were (a) being a nurse or physician providing direct patient care; (b) having at least one year of clinical experience; and (c) having prior experience of collaborating with an intercultural mediator in clinical practice. No exclusion criteria were applied.

Nurse managers and unit directors were contacted and informed about the study aims and procedures. They were asked to disseminate study information within their units to identify eligible professionals. Healthcare professionals meeting inclusion criteria were invited to contact the researchers directly (A.A. and G.C.) to express their willingness to participate. This recruitment approach ensured voluntary participation, minimized potential hierarchical influence, and guaranteed that declining or withdrawing from the study had no consequences for participants’ professional roles.

A total of 13 healthcare professionals (9 nurses and 4 physicians) participated. Purposive sampling was complemented by snowball sampling to facilitate access to professionals across different clinical areas and work settings. The sample included participants from different professional and clinical backgrounds, enabling the exploration of diverse perspectives [28]. Sample adequacy was considered using the information power approach proposed by Malterud et al. [29]. The sample was considered appropriate for the exploratory and descriptive aim of the study because participants were purposively selected among healthcare professionals with direct experience of collaborating with intercultural mediators, and the interviews provided rich and relevant data for addressing the research question. The cross-case analysis across professional and clinical contexts further supported the adequacy of the overall sample. However, given the limited number of physicians included, sample adequacy was considered at the overall sample level and was not claimed separately for each professional subgroup.

### 2.3. Data Collection

Data were collected between June and July 2024. Semi-structured, face-to-face interviews were conducted in Italian by two trained research assistants (D.I. and L.A.), one male and one female, both doctoral candidates in Nursing Science and Public Health who also work as registered nurses. Both had a background in clinical nursing and training in qualitative interviewing. The interviewers had no prior relationship with participants.

Before the interviews, participants were informed about the researchers’ academic background, their doctoral training, and the study’s aim to explore experiences of collaboration with intercultural mediators in clinical practice. The interviewers introduced themselves as researchers interested in improving understanding of intercultural care processes and clarifying the organizational role of IMs within healthcare settings.

Given their professional background as nurses, the interviewers acknowledged the potential influence of their clinical perspectives on data interpretation. Reflexive awareness was maintained throughout data collection and analysis, and regular research team discussions were conducted to examine assumptions and minimize interpretive bias.

Interviews were conducted in private rooms within the hospital setting to ensure confidentiality and minimal interruption. Interview timing and location were agreed upon with participants to accommodate clinical responsibilities and promote comfort. A semi-structured interview guide (Supplementary File S2) supported consistency while allowing for flexibility to elicit detailed accounts of participants’ experiences. Field notes were recorded during and immediately after each interview to document non-verbal cues and contextual information.

Interviews were audio-recorded using a digital device and lasted approximately 30–45 min. Participants provided demographic information (gender, age, profession, years of clinical experience) to support contextual description and assessment of transferability.

### 2.4. Data Analysis

Data analysis was conducted using inductive qualitative content analysis, following the principles described by Elo and Kyngas (2008) [30]. Individual interviews were treated as units of analysis. The analytic process involved three interrelated phases: open coding, categories creation, and abstraction.

During the open coding phase, transcripts were read repeatedly and carefully, and notes and headings were written alongside the text to capture all relevant aspects of the content. Codes were generated using words or expressions that closely reflected participants’ own language, while preserving the overall meaning of their statements. During the categories creation phase, codes were examined and compared, and those sharing conceptual or semantic similarities were grouped into broader subcategories. In the abstraction phase, subcategories with conceptual and semantic similarities were grouped into generic categories.

Two researchers (A.A. and G.C.) independently read all interviews line by line and extracted codes, subcategories, and categories. A senior qualitative researcher (DI) reviewed and compared the codes and categories identified based on the transcripts. Disagreements were resolved through team discussion until consensus was reached. To enhance transparency, the analytic process was documented through an audit trail, including coding notes, preliminary groupings, and iterative revisions of the coding framework. Initial coding generated 25 codes, which were then organized into six subcategories and three main categories. A coding matrix linking categories, subcategories, participant sources, and illustrative quotations was developed and is provided in Supplementary File S3.

Data collection and analysis proceeded concurrently and continued until no new meaningful information, codes, or categories emerged from subsequent interviews [31]. The research team determined that sufficient informational redundancy had been reached, indicating data saturation, when additional interviews no longer generated substantially new codes or meaningful variations in the developing categories. This occurred during the 12th interview; however, researchers conducted one additional interview to confirm code stability. Saturation was assessed at the overall sample level, evaluating the recurrence and stability of the main categories across participants and clinical contexts. All analytic procedures were performed manually.

### 2.5. Ethical Considerations

The study adhered to the ethical principles of the Declaration of Helsinki [32]. Ethical approval was obtained from the local Institutional Review Board Rome-Lazio, Italy (No. 0013672/24 on 15 April 2024). Participants received clear and comprehensive information about the study’s aims, procedures, potential risks and benefits, data protection measures, and voluntary nature of participation. Informed consent, including consent to audio recording and anonymous data use, was obtained from all participants. Participants were assigned identification codes, and all transcripts were de-identified. Data were securely stored and accessible only to the research team. Participants were informed of their right to withdraw at any time without consequences.

### 2.6. Trustworthiness

Trustworthiness was ensured in line with the criteria of credibility, dependability, confirmability, and transferability Lincoln and Guba [33] and with the principle of ongoing methodological verification [34].

Credibility was supported through prolonged engagement with the data and regular debriefings within the research team. These discussions enabled researchers to compare interpretations and examine potential preconceptions, supporting reflexivity throughout the analytic process.

Dependability and confirmability were ensured through use of the audit trail. Two researchers, not involved in data collection and analysis, examined the process and the outcomes of the inquiry to ensure that the findings were supported by data. In addition, the process was further supervised by a senior researcher with expertise in qualitative methods who was not directly involved in data collection.

Transferability was supported by providing a detailed description of the study context, participants’ sociodemographic characteristics, and the research process. By offering a rich and contextualized account of the findings, this study enables readers to assess the relevance and applicability of the results to similar healthcare settings involving intercultural mediation.

## 3. Results

### 3.1. Characteristics of Participants

Sociodemographic and professional characteristics are summarized in Table 1.

The sample included 13 healthcare professionals, 9 nurses (69.2%) and 4 physicians (30.8%), working across different clinical settings. The mean age was 41.3 years (SD = 10.0); most participants were female (n = 11, 84.6%). The average professional experience was 10.5 years (SD = 8.0), ranging from 1 to 25 years among nurses (median = 15 years) and from 2 to 8 years among physicians (median = 4.5 years). Detailed participant information is summarized in Table 2.

### 3.2. Categories

Three main categories emerged from the analysis (Table 3): (1) the experience of professionals in the relationship with migrant patients; (2) the linguistic and intercultural mediator as a supportive relational role; (3) limitations in language and intercultural mediation (Supplementary File S3). Together, these categories describe intercultural mediation as a relational bridge that facilitates understanding and trust, while also revealing organizational constraints that limit its full integration in care processes.

Representative quotations supporting each category and subcategory are reported in the text and further detailed in Supplementary File S3.

#### 3.2.1. Category 1: The Experience of Healthcare Professionals in the Relationship with Migrant Patients

This category describes health professionals’ experiences in their encounters with migrant patients and highlights the IM’s role in facilitating mutual understanding between different cultural worlds. Two subcategories emerged: (1) the IM as a bridge between different cultural worlds, and (2) the challenge of achieving mutual understanding.

##### Subcategory 1.1: The Intercultural Mediator as a Bridge Between Different Cultural Worlds

Participants described IMs as more than translators, emphasizing their role in interpreting culturally embedded meanings and behaviours:

*“That’s why we have cultural mediators, who are not mere translators. They are people who often come from those countries and help us understand not only the language, but also what lies behind certain attitudes or ways of behaving. Without them, many situations would be much more difficult to interpret”*.

*(RN09)*

IMs were seen as helping professionals contextualize behaviours that might otherwise be misunderstood:

*“Here there is always someone, a mediator or sometimes an anthropologist, who can explain why a patient behaves in a certain way. It’s not just a matter of words, but of understanding the meaning of what we see and what happens during the encounter”*.

*(RN03)*

However, some participants suggested that adapting care to cultural perspectives often depends on individual professionals rather than being structurally supported:

*“If we understand ‘cultural mediation’ as also including the ability to adapt to the patient’s culture, this aspect is often neglected and left entirely to the goodwill of individual professionals, most often nurses…”*.

*(P01)*

##### Subcategory 1.2: The Challenge of Achieving Mutual Understanding

Despite mediation support, interpretive difficulties persist due to the complexity of patients’ experiences and worldviews:

*“I was struck by a boy, Indian or Pakistani, who was talking to me but wouldn’t look me in the face. At first, it made me uncomfortable because I didn’t know how to interpret that behavior. Situations like this make you realize how easy it is to misunderstand if you don’t have cultural references”*.

*(RN08)*

These accounts portray healthcare professionals lived experience as marked by ongoing uncertainty and a tension between the intention to communicate and the ability to fully grasp culturally embedded meanings, gestures and reactions. Intercultural mediation is therefore experienced not merely as a technical resource, but as essential relational support in encounters that challenge habitual clinical interpretations. Such situations highlight both the value of mediation in everyday practice and the inherent limits of cross-cultural understanding in clinical settings.

This interpretive challenge is further reinforced by another professional, who emphasizes that linguistic translation alone is insufficient to grasp culturally embedded meanings:

*“…In my opinion, what they are doing is very important, but it is clear that language translation alone is not enough, because a gesture may have a different meaning in different cultures. For example, the word ‘hip’ may not refer to the same area in all cultures…”*.

*(P03)*

#### 3.2.2. Category 2: The Linguistic and Intercultural Mediator as a Supportive Relational Role

This category highlights the value attributed to IMs in fostering trust, safety and effective communication.

Two subcategories emerged: (1) trust and safety in the relationship, and (2) professionals’ reflection on the value of IM within the care process.

##### Subcategory 2.1: Trust and Safety in the Relationship

Participants emphasized how mediation supports respectful communication without compromising patients’ beliefs:

*“Yes, thanks to the intervention of IMs we were able to explain the importance of care without violating their beliefs. Without mediation, these situations would be much more difficult to manage, because patients might feel misunderstood or disrespected”*.

*(RN09)*

IMs were described as facilitating understanding of biomedical procedures within culturally different conceptions of health:

*“Migrant patients’ concept of health is definitely different from ours. The positive thing here is that there are facilitators who speak the same language and help them understand why, for example, we do certain procedures, like blood tests. This makes communication much easier and helps patients accept what we are doing”*.

*(RN03)*

##### Subcategory 2.2: Reflection on the Value of Intercultural Mediation

Participants reported that mediation enhances personalization of care and supports patient comprehension:

*“In this hospital I have noticed that nurses and IMs really try to make the patient understand everything. Before leaving the hospital, the patient has to understand what they have to do or what has been done -everything. I haven’t seen this level of attention in hospitals where IMs are not present”*.

*(RN04)*

The availability of mediation services was also described as reassuring at both clinical and organizational levels:

*“…It is reassuring for me to know that this service exists. I cannot imagine how they managed before when mediators did not exist…”*.

*(P04)*

Trust was described as developing through mediated encounters that acknowledge patients’ beliefs and expectations. Some participants also noted that mediation strengthens connections with the broader community and supports continuity of care:

*“…Empowering the territory, including through cultural mediation…”*.

*(P04)*

Overall, IMs were perceived as essential relational actors who integrate clinical planning with attentive listening to patients’ lived experiences.

#### 3.2.3. Category 3: Limitations in Language and Intercultural Mediation

This category described intercultural mediation as constrained by organizational and structural limitations.

Two subcategories emerged: (1) organizational limits and limited availability of mediation services, and (2) poor integration of the intercultural mediator’s role within healthcare teams.

##### Subcategory 3.1: Organizational Limits and Limited Availability

Participants frequently reported insufficient availability of mediators during critical phases of care:

*“Mediators are great, but they have shifts too. It can happen that, for example, last week one afternoon the mediator was not there, and we had to manage using mobile phones. In those situations, communication becomes much more complicated”*.

*(RN02)*

In high-intensity settings such as emergency departments, this absence was perceived as particularly problematic:

*“…These families need a set of guidelines when they arrive at the emergency room… they need someone to explain how the emergency services works…”*.

*(P01)*

Overall, limited access to mediation services was seen as undermining continuity and increasing the risk of misunderstandings.

##### Subcategory 3.2: Poor Integration of the Intercultural Mediator’s Role Within the Team

Beyond availability, participants described mediators as occupying a peripheral position within healthcare teams. Mediation was often activated reactively rather than embedded throughout the care pathway:

*“It’s an excellent service, but the way it is organized means that the mediator remains external to the team. They often don’t really know our daily work and are not involved in decision-making processes”*.

*(P01)*

Some participants perceived mediators primarily as translators rather than integrated team members:

*“It’s an excellent service, but for me the mediator is outside the team, has nothing to do with our work, and acts as a translator”*.

*(P03)*

This limited integration was described as leading to underutilization:

*“We tend to underestimate the mediator’s role, because we use it mainly just to ensure basic understanding and not in a comprehensive way throughout the care process”*.

*(P02)*

Healthcare professionals expressed frustration about this “on-call” model of mediation, which was perceived as insufficient to support relational continuity in multicultural care settings.

## 4. Discussion

This qualitative descriptive study aimed to explore the experiences of healthcare professionals in collaborating with IMs in the context of caring for migrant patients. Three main categories emerged, highlighting both the relational value of IMs and the structural limitations affecting their integration. These findings align with the growing European literature, suggesting that encounters in healthcare are often marked by communicative complexity and potential misunderstanding.

Although no formal subgroup analysis was conducted, some contextual differences were observed across professional accounts. Nurses more often emphasized trust-building, relational continuity, explanation of care, and patient reassurance. Physicians more frequently referred to role boundaries, organizational issues, and the limited involvement of IMs in decision-making processes. These observations should be interpreted cautiously, as descriptive insights rather than comparative findings.

Consistent with previous research [35,36,37], IMs were described as essential actors in enhancing mutual understanding and fostering culturally responsive care. Their contribution extended beyond linguistic translation to include bridging sociocultural expectations, clarifying culturally embedded health beliefs, and supporting equitable care delivery [36,37]. Participants perceived IMs as facilitating clearer communication, improving patients’ understanding of treatment plans, and reducing professional frustration during cross-cultural consultations.

This broader interpretive role is reflected in qualitative findings from Germany, where IMs are conceptualized as cultural brokers who mitigate emotional strain and support trust-building in ethically complex encounters. Similarly, Baraldi (2017) highlights how intercultural mediation can reduce interpersonal tension and support smoother clinical interactions by clarifying expectations and cultural norms [38].

Spanish research also highlights how IMs are positioned as key navigators within the healthcare system, accompanying patients within and beyond the healthcare system, adapting written medical materials, and orienting them within institutional processes [39].

These professional impressions underscore that IMs are seen not merely as linguistic aides but as co-navigators of intercultural complexity, enabling smoother workflows, relational trust, and professional reassurance in multicultural healthcare environments [40].

However, despite their recognized value, IMs were perceived as insufficiently integrated within healthcare teams. Participants described their involvement as peripheral and episodic and reactive rather than structurally embedded. Limited availability, unclear mandates, and absence of standardized role definitions constrained their capacity to contribute to continuous, culturally competent care. Similar tensions have been reported by Anzini et al. (2025) where role ambiguity and fragmented institutional recognition hindered effective collaboration [41].

Healthcare professionals often misunderstand or underestimate the scope of intercultural mediation, conflating it with linguistic translation alone [35]. This confusion is echoed in another research in a northern Italian context where healthcare staff show divergent expectations from mediator’s functions, some requesting only linguistic translation, while others relying on IMs for complex social and relational tasks [22]. Such ambiguity can generate professional tensions, particularly when IMs advocate for culturally informed patient needs that may challenge clinicians’ authority or ethical boundaries, leading to discomfort or resistance among staff [36]. Comparable difficulties in trust-building between IMs and clinical teams have also been observed in Belgium, especially in large institutional settings lacking relational continuity [42].

Across European contexts, the absence of standardized legal and organizational frameworks contributes to IMs operating without formal guidelines or institutionalized roles [22,35,36]. Although valued for their immediate communicative functions, IMs are rarely embedded within clinical decision-making structures in a way that enables sustained collaborative and systemic impact [12,36].

This limited integration not only limits the potential contribution of IMs but also the structural foundations required to deliver culturally appropriate care.

In Italian specifically, institutional recognition of IMs remains fragmented. Despite formal acknowledgment in Law No. 40 of March 6, 1998, no comprehensive regulatory framework has standardized training pathways, professional registers, or integration mechanisms [22].

Misalignment between national policies and local implementation further contributes to variability and uncertainty regarding competencies and responsibilities, resulting in the absence of coordinated strategies for mediator integration [43,44]. Consequently, IMs are frequently conflated with interpreters or social workers, weakening their professional identity and limiting their strategic positioning within healthcare organizations.

While the role of IMs is firmly established and institutionally recognized in healthcare systems across the United States, European systems, by contrast, continue to show significant gaps in both recognition and implementation. As observed across various qualitative studies in Italy, Germany, Spain, Belgium, and the Czech Republic, there is a pressing need to promote greater organizational integration of intercultural mediation services and to foster intercultural competence among healthcare professionals [22,35,36,39,42].

The present study reinforces these findings, indicating that the limited integration of IMs is compounded by a general lack of preparedness among healthcare staff to engage meaningfully in intercultural dynamics. Therefore, incorporating intercultural training into the professional development of healthcare professionals is critical. Such training not only enhances individual knowledge and skills but also strengthens collaboration between clinicians and IMs, thereby fostering more effective and inclusive care for migrant populations [22].

Targeted training interventions allow staff to progress from unconscious incompetence to conscious competence, increasing their awareness of cultural dynamics and enabling them to recognize when and how to involve IMs appropriately [45]. Moreover, this preparedness promotes the perception of the intercultural mediator as a legitimate and integral member of the care team, capable of contributing meaningfully at all stages of the patient’s journey—from initial admission to discharge and community follow-up.

Compared with the more institutionalized recognition observed in parts of the United States, European systems continue to display gaps in formal integration and policy support. These structural limitations appear intertwined with a broader lack of intercultural preparedness among healthcare professionals [42].

Further qualitative and quantitative research in the Italian context is needed to assess the actual availability and functionality of intercultural mediation services, including around-the-clock access and structured integration mechanisms within care teams. Future studies should also broaden the participant base beyond doctors and nurses to include allied health professionals, healthcare managers, and policy stakeholders. In particular, research should examine how IM characteristics, such as cultural and linguistic background, gender, age, professional training, prior experience, and familiarity with specific clinical settings, may influence trust-building, perceived legitimacy, and collaboration with both patients and healthcare professionals.

Ultimately, IMs represent an important resource for culturally responsive care, but their contribution alone is not sufficient. Systemic commitment, institutional investment, and intercultural capacity-building at all levels remain critical to fostering truly inclusive healthcare environments.

### 4.1. Strengths and Limitations

One of the key strengths of this study lies in its contribution to a relatively underexplored area in the Italian context, the perceptions and experiences of healthcare professionals in their collaboration with IMs. By focusing on this dimension of healthcare provision, the research offers valuable insights into how intercultural mediation is understood and operationalized within the broader system of migrant care. Our study highlighted how the role of the intercultural mediator is fragmented in Italy and how its presence is not homogeneous in different contexts.

However, several limitations should be acknowledged. First, the research was conducted only in two geographical areas of Italy, which may limit the transferability of findings to regions with different healthcare infrastructures, migration patterns, and service coordination models. Second, although sample adequacy was supported by the information power of participants’ accounts and by the specificity of the study aim, the sample remained relatively small. In particular, the physician subgroup included only four participants, all of whom were early-career professionals. Therefore, the findings may not fully capture the perspectives of mid-career or senior physicians, who may experience collaboration with IMs differently. Third, data saturation was assessed across the overall sample rather than separately within professional groups; therefore, physician-specific insights should be interpreted with caution. Finally, this study explored only healthcare professionals’ views. Future research should adopt multi-perspective approaches, including other healthcare actors, including relevant stakeholders, social workers, administrative personnel, and migrant patients themselves. Such approaches could offer a more comprehensive view of the systemic enablers and barriers affecting the integration of IMs in healthcare teams.

### 4.2. Implications for Clinical Practice

At the government and policymaker level, the implementation of existing legislation through clearer regulatory decrees would help define the role, training pathways and organizational positioning of IMs. At local level, healthcare organizations should move beyond viewing IMs as peripheral actors and instead include them in care planning, patient consultations, and multidisciplinary discussions to foster culturally safe, patient-centred communication. Healthcare staff should receive targeted intercultural training to enhance professionals’ ability to address culturally informed health beliefs. It would be appropriate to include teaching and awareness of the importance of cultural mediators in university curricula. Clear institutional guidelines are needed to define the IMs’ roles, reduce ambiguity and prevent role-related tensions. Ensuring timely and continuous access to mediation services—especially for vulnerable groups—is crucial for promoting equitable care [46]. Continuity in assigning the same IM across care encounters may strengthen trust and communication consistency. Routine monitoring and evaluation of mediation services, using defined indicators, can support service adaptation and organizational learning.

## 5. Conclusions

This study suggests that, from healthcare professionals’ perspectives, IMs play an important but still insufficiently integrated role in supporting culturally responsive care for migrant patients in the Italian hospital contexts explored. IMs are perceived as relational bridge-builders who facilitate communication, foster trust and help navigate sociocultural complexity within clinical encounters. However, their contribution remains limited by fragmented policies, a lack of standardized training, and limited integration within healthcare teams. Without stable institutional embedding, the potential of intercultural mediation to enhance continuity, equity and quality of care remains only partially realized.

Strengthening the organizational embedding of intercultural mediation and integrating intercultural competence into professional development are essential steps toward more equitable and inclusive healthcare systems. Future research is needed to explore the structural, professional, and mediator-related conditions that enable sustainable and effective mediation practices across diverse healthcare contexts.

## Figures and Tables

**Table 1:** Characteristics of participants (N = 13).

Characteristics	
	M (±SD)
Age	41.3 (10.0)
Professional experience (years)	10.5 (8.0)
	Median; Range (min–max)
Nurse	15 (1–25)
Physician	4.5 (2–8)
	N (%)
Gender identity	
Woman	11 (84.6)
Man	2 (15.4)
Profession	
Nurse	9 (69.2)
Physician	4 (30.8)

Legend. M, Mean; N, Number; SD, Standard Deviation; Min, Minimum; Max, Maximum.

**Table 2:** Detailed participant information.

ID	Age	Gender	Years of Experience	Profession
RN01	43	Woman	20	Nurse
RN02	56	Woman	23	Nurse
RN03	46	Woman	15	Nurse
RN04	26	Woman	1	Nurse
RN05	46	Woman	15	Nurse
RN06	42	Woman	9	Nurse
RN07	51	Woman	14	Nurse
RN08	49	Woman	25	Nurse
RN09	52	Man	15	Nurse
P01	32	Woman	5	Physician
P02	28	Woman	2	Physician
P03	31	Man	4	Physician
P04	33	Woman	8	Physician

Note: RN = Interviewee (nurse); P = Interviewee (physician). Numerical codes (e.g., RN01, P01) indicate anonymized participant identifiers.

**Table 3:** Main categories and sub-categories.

Main Categories	Subcategories	Source
1. The experience of professionals in the relationship with the migrant patient	The mediator: a bridge between different cultural worlds	RN09, RN03, RN01, RN08, P01, P02
	The challenge in mutual understanding	RN08, P03, P04
2. The Linguistic and Intercultural Mediator as a Supportive Relational Figure	Trust and safety in the relationship	RN09, RN03, RN02
	Reflection on the value of intercultural mediation	RN04, RN07, RN08, RN09, P04
3. Limitations in language and intercultural mediation	Organizational limits and limited availability	RN02, RN05, P01, P02, P03
	Poor integration into the team	RN02, RN05, P01, P02, P03

Note: RN = Interviewee (nurse); P = Interviewee (physician). Numerical codes (e.g., RN01, P01) indicate anonymized participant identifiers.

## Data Availability

The data presented in this study are available on reasonable request from the corresponding author due to privacy restrictions.

## Supplementary Materials

The following supporting information can be downloaded at: https://www.mdpi.com/article/10.3390/healthcare14131903/s1, File S1: COREQ Checklist; File S2: Semi-Structured Interview Guide; File S3: Categories and Subcategories.

## References

[1] Dao T.H., Docquier F., Maurel M., Schaus P. (2021). Global migration in the twentieth and twenty-first centuries: The unstoppable force of demography. Rev. World Econ..

[2] Ozden C., Parsons C.R., Schiff M., Walmsley T.L. (2011). Where on Earth is Everybody? The Evolution of Global Bilateral Migration 1960–2000. World Bank Econ. Rev..

[3] Wesolowska K., Hietapakka L., Elovainio M., Aalto A.M., Kaihlanen A.M., Heponiemi T. (2018). The association between cross-cultural competence and well-being among registered native and foreign-born nurses in Finland. PLoS ONE.

[4] ISTAT (2025). Internal and International Migration of the Resident Population. Years 2023–2024.

[5] Alarcon F.J. (2022). The Migrant Crisis and Access to Health Care. Dela J. Public Health.

[6] Heath M., Hvass A.M.F., Wejse C.M. (2023). Interpreter services and effect on healthcare—A systematic review of the impact of different types of interpreters on patient outcome. J. Migr. Health.

[7] Díaz-Millón M., Olvera-Lobo M.D. (2025). Systematic meta-review on migrant healthcare access: Language barriers and the role of translation. J. Migr. Health.

[8] Aldin A., Baumeister A., Chakraverty D., Monsef I., Noyes J., Kalbe E., Woopen C., Skoetz N. (2024). Gender differences in the context of interventions for improving health literacy in migrants: A qualitative evidence synthesis. Cochrane Database Syst. Rev..

[9] Kerr D., Martin P., Furber L., Winterburn S., Milnes S., Nielsen A., Strachan P. (2022). Communication Skills Training for Nurses: Is It Time for a Standardised Nursing Model?. Patient Educ. Couns..

[10] Shahzad S., Ali N., Younas A., Tayaben J.L. (2021). Challenges and approaches to transcultural care: An integrative review of nurses’ and nursing students’ experiences. J. Prof. Nurs..

[11] IOM, International Organization for Migration (2024). Migration and Migrants: Regional Dimensions and Developments. World Migration Report 2024.

[12] Verrept H. (2019). What Are the Roles of Intercultural Mediators in Health Care and What Is the Evidence on Their Contributions and Effectiveness in Improving Accessibility and Quality of Care for Refugees and Migrants in the WHO European Region?.

[13] Pokorn N.K., Mikolič Južnič T. (2020). Community interpreters versus intercultural mediators. Transl. Interpret. Stud..

[14] Phelan M., Martín M. (2010). Interpreters and cultural mediators—Different but complementary roles. Translocations.

[15] Verrept H., Valero-Garcés C., Martin A. (2008). Intercultural mediation: An answer to healthcare disparities?. Crossing Borders in Community Interpreting.

[16] Guionnet A., Estevez L., Navaza B., Navarro M. (2009). Intercultural mediation as solution to linguistic and cultural conflicts between health personnel and migrant patients and a way to social integration. Trop. Med. Int. Health.

[17] Taglieri F., Colucci A., Barbina D. (2013). Communication and cultural interaction in health promotion strategies to migrant populations in Italy: The cross-cultural phone counselling experience. Ann. dell’Istituto Super. Sanità.

[18] Škraban J., Oprešnik D., Pistotnik S., Čebron U.L. (2020). Implementation of intercultural mediation at the primary level in preventive healthcare in Slovenia VPELJEVANJE MEDKULTURNE MEDIACIJE V PREVENTIVNE PROGRAME NA PRIMARNEM NIVOJU ZDRAVSTVA V SLOVENIJI. Javno Zdr..

[19] Alcaraz Quevedo M., Paredes-Carbonell J.J., Sancho Mestre C., López-Sánchez P., García Moreno J.L., Vivas Consuelo D. (2014). Immigrants women care in a health intercultural mediation program. Rev. Esp. Salud Publica.

[20] Miklavcic A., LeBlanc M.N., Kirmayer L., Guzder J., Rousseau C. (2014). Culture Brokers, Clinically Applied Ethnography, and Cultural Mediation. Cultural Consultation. International and Cultural Psychology.

[21] Ministry of Education, Universities, and Research (2007). Ministerial Decree of March 16, 2007—Determination of University Degree Classes.

[22] De Leo A., Ceschi A., Mutti M. (2024). The Cultural Mediator as a Facilitator for Collaboration: Fostering Inter-Institutional Networking Among Services for Forced Migrants. Informing Sci. Int. J. Emerg. Transdiscipl..

[23] ISMU Foundation ETS (2024). 30th Report on Migration 2024.

[24] Vandecasteele R., Robijn L., Stevens P.A.J., Willems S., De Maesschalck S. (2024). “Trying to write a story together”: General practitioners’ perspectives on culturally sensitive care. Int. J. Equity Health.

[25] García-Navarro E.B., da Costa E.M.T. (2017). Intercultural Mediation at the End of Life. Different Perceptions of the Same Process. Procedia—Soc. Behav. Sci..

[26] Sandelowski M. (2000). Whatever happened to qualitative description?. Res. Nurs. Health.

[27] Tong A., Sainsbury P., Craig P. (2007). Consolidated criteria for reporting qualitative research (COREQ): A 32-item checklist for interviews and focus groups. Int. J. Qual. Health Care.

[28] Polit D.F., Beck C.T. (2008). Nursing Research: Generating and Assessing Evidence for Nursing Practice.

[29] Malterud K., Siersma V.D., Guassora A.D. (2016). Sample Size in Qualitative Interview Studies: Guided by Information Power. Qual. Health Res..

[30] Elo S., Kyngas H. (2008). The qualitative content analysis process. J. Adv. Nurs..

[31] Naeem M., Ozuem W., Howell K., Ranfagni S. (2024). Demystification and Actualisation of Data Saturation in Qualitative Research Through Thematic Analysis. Int. J. Qual. Methods.

[32] WMA WMA Declaration of Helsinki—Ethical Principles for Medical Research Involving Human Participants. https://www.wma.net/policies-post/wma-declaration-of-helsinki/.

[33] Lincoln Y.S., Guba E.G. (1985). Naturalistic Inquiry.

[34] Morse J.M., Barrett M., Mayan M., Olson K., Spiers J. (2002). Verification strategies in qualitative research. Int. J. Qual. Methods.

[35] Těšinová J.K., Dobiasova K., Jelinkova M., Tulupova E., Koscik M. (2024). Professionals’ and intercultural mediators’ perspectives on communication with Ukrainian refugees in the Czech healthcare system. Health Expect..

[36] Luetke Lanfer H., Touzel V., Reifegerste D. (2025). Semi-professional language mediators in patient–provider interactions in Germany: An interview study. BMC Health Serv. Res..

[37] Anderson L.J. (2021). The emergence and relevance of cultural difference in mediated health interactions. Health Commun..

[38] Baraldi C., Gavioli L. (2017). Intercultural Mediation and (Non)Professional Interpreting in Italian Healthcare Institutions.

[39] Ribas M.A. (2017). The fuzzy boundary between the roles of interpreter and mediator in the public services in Catalonia: Analysis of interviews and interpreter-mediated interactions in the health and educational context. Across Lang. Cult..

[40] Caggianelli G., Cangelosi G., Dello Iacono I., Petrelli F., Fiorda M., Pettinari S., Palomares S.M., Marfella F., Mancin S. (2025). The experience of volunteer nurses providing health and social support to refugees during the war in Ukraine: A phenomenological study. Int. J. Nurs. Stud. Adv..

[41] Anzini A., Caggianelli G., Tomasin R.P., Segala A., Giangrazi S., Paccagnella C., Mancin S., Petrelli F., Vanzi V., Cangelosi G. (2025). Transcultural challenges in the nurse-migrant patient caring relationship: A phenomenological study. Appl. Nurs. Res..

[42] Van Keer R.L., Fernandez S.M., Bilsen J. (2020). Intercultural mediators in Belgian hospitals: Demographic and professional characteristics and work experiences. Patient Educ. Couns..

[43] Cavallari C.C., Avolio G.M. (2024). The blurred lines of intercultural mediation: Professional recognition through formal and informal practices in Italy. Europa.

[44] Genova A.B. (2018). Social workers and intercultural mediators: Challenges for collaboration and intercultural awareness. Eur. J. Soc. Work.

[45] Macmillan M. (1970s). The Journey from Unconscious Incompetence to Unconscious Competence Adapted from Noel Birch. https://www.macmillancentreforlearning.co.uk/the-journey-from-unconscious-incompetence-to-unconscious-competence-adapted-from-noel-birch-1970s-by-malcolm-macmillan/.

[46] Handtke O., Schilgen B., Mosko M. (2019). Culturally competent healthcare: A scoping review of strategies implemented in healthcare organizations and a model of culturally competent healthcare provision. PLoS ONE.

