# Supplementary material for: The Intercultural Mediator as a Bridge in Healthcare Professional–Migrant Patient Care Relationships: A Qualitative Study
Source: Healthcare (Basel). 2026 Jun 30;14(13):1903. doi: 10.3390/healthcare14131903 (PMC13360903; doi:10.3390/healthcare14131903)
Supplement: Supplementary file 1 [file healthcare-14-01903-s001.zip › 3_V2.pdf]

3. Main categories and Sub-categories:

Main Categories	Sub-Categories	Codes	Sentences of Meaning	Source
1. Experience of healthcare professionals in the relationship with migrant patients	The mediator: a bridge between different cultural worlds	Mediator as more than translator	"…That's why we have cultural mediators, who are not mere translators, but are people who often come from those very countries..."	RN09
		Cultural interpretation of patient behaviour	"…Here there is always someone (mediator or anthropologist) who can explain to you why a patient behaves a certain way..."	RN03
		Mediator helps reframe assumptions	"…With one woman, I thought one thing, I thought she was under her husband's thumb in wanting to have children, but in reality, it was she who wanted to have children right away, she couldn't have them after a month of trying. So the mediator explained a few things to me, and then we came to an understanding with the patient…"	RN01
		Mediation as support in care process navigation	"…I call the mediator because the problem is not only linguistic but also concerns the process here in Italy. Without mediators, even doctors refuse to perform examinations…"	RN08
		Cultural adaptation left to professionals' goodwill	"…"If we understand 'cultural mediation' as also including the ability to adapt to the patient's culture, this aspect is often neglected and left entirely to the goodwill of individual professionals, most often nurses…"	P01
		Need for deeper intercultural dialogue	"…A more in-depth discussion between the non-EU citizen and the family pediatrician…"	P02
	The challenge in mutual understanding	Difficulty interpreting non-verbal behaviour	"…I was then struck by a boy, Indian or Pakistani, who was talking to me and not looking at my face..."<br><br>"…They have the fear of not being understood, and this shapes the way they approach us. At first, I did not understand them; with experience you gradually learn who you have in front of you and what their state of mind is…"	RN08

		Limits of language translation alone	"…In my opinion, what they are doing is very important, but it is clear that language translation alone is not enough. because a gesture may have a different meaning in different cultures. for example, the word "hip" may not refer to the same area in all cultures…"	P03
		Risk of misunderstanding clinical history	"…In my opinion, we use them too little. we think we understand each other with our essential communications, but a parent deserves much more explanation when their child is sick in the emergency room, you know how worried they are... we also need it to understand the medical history, we could make the wrong diagnosis and prescribe the wrong treatment if we don't understand what the mother is trying to tell us because we could interpret it differently..."	P04
2. The Linguistic and Intercultural Mediator as a Supportive Relational role	Trust and safety in the relationship	Mediation enables respectful care	"…Yes, but thanks to the intervention of mediators we were able to explain the importance of care without violating their beliefs..."	RN09
		Mediator supports understanding of procedures	"…Definitely their concept of health is different from ours. The good thing here is that there are the facilitators who speak the same language, who help them understand why, for example, we do the withdrawal..."	RN03
		Mediation reduces fear and mistrust	"…They are almost terrified of us nurses; although, on the positive side, there are mediators who speak the same language and help them understand why, for example, we take blood samples; because often, especially in African countries, they think we are selling the blood…"	RN03
		Absence of mediator compromises informed understanding	"…A woman came in with her medical records and didn't know that in this hospital, where she had once been admitted as an emergency case, they had removed her uterus... The problem was that she wanted to have children... In the end, it turned out that the medical staff had told her that she had undergone emergency surgery, but she didn't understand properly. This is a mistake on the part of the hospital, because there are no mediators…"	RN02

	Reflection on the value of intercultural mediation	Mediation improves patient understanding at discharge	"…In this hospital I have noticed that they try to make the patient (nurses and mediators) understand everything, before leaving (the hospital) the patient has to understand what they have to do or what they have done, everything! Which I have not seen in other hospitals without mediators…"<br><br>"…If there is no mediator, we try to make ourselves understood. For example, if I have to take a blood sample, I use gestures to show what the patient has to do; sometimes I also use Google Translate…"	RN04
		Mediators as patient reference point	"…mediators are also a point of reference for patients…"<br>"…One becomes a point of reference, and even after patients obtain their residence permit, they still come back here…"	RN07
		Mediation supports access to care pathways	"…The problem is not only linguistic, but also concerns the process here in Italy. Without mediators, even doctors refuse to perform examinations…"	RN08
		Patients may hide lack of understanding	"…A lot of times they say yes so as not to offend, because they have so much respect for the uniform; a lot of times they say yes out of fear; a lot of times they say yes out of shame. You know what I mean? Yeah. They don't understand, but they say yes because they're ashamed to tell you they don't understand. That's why we have cultural mediators…"	RN09
		Presence of mediation service as reassurance	"…It is reassuring for me to know that this service exists. I cannot imagine how they managed before when mediators did not exist…"	P04
		Mediation strengthens community care links	"…Empowering the territory, including through cultural mediation…"	P04
3. Limitations in language and	Organizational limits and	Mediator unavailable during care needs	"…Mediators are great, but they have shifts too; it can happen that, for example last week, one afternoon the mediator was not there and we got busy with cell phones..."	RN02

intercultural mediation	limited availability		"…The mediators have shifts too; when one afternoon the mediator was not there, we managed with a mixture of English and French, trying to make ourselves understood…"	
		Need for more mediation resources	"…We need more cultural mediators... there should be more resources, more nurses..."	RN05
		Need for mediation in emergency access	"…These families need a set of guidelines when they arrive at the emergency room... they need someone to explain how the emergency services works…"	P01
	Poor integration into the team	Mediator perceived as external to team	"…It's a great service, but for me the mediator is external to the team, he doesn't know our work…"	P01
		Mediator role underused in care process	"…We underestimate it, because we use it only for understanding, but we don't use it in the round, we use it only in a small part..."	P02
		Mediator reduced to translator role	"…It's an excellent service, but for me the mediator is outside the team, has nothing to do with our work, and acts as a translator…"	P03

Note: RN= Interviewee (nurse); P= Interviewee (physician). Numerical codes (e.g., RN01, P01) indicate anonymised participant identifiers.
