# Supplementary material for: The Intercultural Mediator as a Bridge in Healthcare Professional–Migrant Patient Care Relationships: A Qualitative Study
Source: Healthcare (Basel). 2026 Jun 30;14(13):1903. doi: 10.3390/healthcare14131903 (PMC13360903; doi:10.3390/healthcare14131903)
Supplement: Supplementary file 1 [file healthcare-14-01903-s001.zip › 1_V2.pdf]

| Topic | Item No. | Guide Questions / Description | Reported on Page No. |
|---|---|---|---|
| **Domain 1: Research Team and Reflexivity** | | | |
| Personal characteristics | | | |
| Interviewer / facilitator | **1** | Which author/s conducted the interview or focus group? | 5 |
| Credentials | **2** | What were the researcher's credentials? E.g. PhD, MD | 5 |
| Occupation | **3** | What was their occupation at the time of the study? | 5 |
| Gender | **4** | Was the researcher male or female? | 5 |
| Experience and training | **5** | What experience or training did the researcher have? | 5 |
| Relationship established | **6** | Was a relationship established prior to study commencement? | 5 |
| Participant knowledge of the interviewer | **7** | What did the participants know about the researcher? e.g. personal goals, reasons for doing the research | 5 |
| Interviewer characteristics | **8** | What characteristics were reported about the interviewer/facilitator? e.g. Bias, assumptions, reasons and interests in the research topic | 5-6 |
| **Domain 2: Study Design** | | | |
| Theoretical framework | | | |
| Methodological orientation and Theory | **9** | What methodological orientation was stated to underpin the study? e.g. grounded theory, discourse analysis, ethnography, phenomenology, content analysis | 4 |
| Participant selection | | | |
| Sampling | **10** | How were participants selected? e.g. purposive, convenience, consecutive, snowball | 5 |

| Topic | Item No. | Guide Questions / Description | Reported on Page No. |
| --- | --- | --- | --- |
| Method of approach | **11** | How were participants approached? e.g. face-to-face, telephone, mail, email | 5 |
| Sample size | **12** | How many participants were in the study? | 5; Tables 1-2, pp. 7-8 |
| Non-participation | **13** | How many people refused to participate or dropped out? Reasons? | N/A |
| Setting | | | |
| Setting of data collection | **14** | Where was the data collected? e.g. home, clinic, workplace | 6 |
| Presence of non-participants | **15** | Was anyone else present besides the participants and researchers? | 6 |
| Description of sample | **16** | What are the important characteristics of the sample? e.g. demographic data, date | 7-8; Tables 1-2 |
| Data collection | | | |
| Interview guide | **17** | Were questions, prompts, guides provided by the authors? Was it pilot tested? | 6; Supplementary File 2 |
| Repeat interviews | **18** | Were repeat interviews carried out? If yes, how many? | N/A |
| Audio/visual recording | **19** | Did the research use audio or visual recording to collect the data? | 6 |
| Field notes | **20** | Were field notes made during and/or after the interview or focus group? | 6 |
| Duration | **21** | What was the duration of the interviews or focus group? | 6 |
| Data saturation | **22** | Was data saturation discussed? | 6 |
| Transcripts returned | **23** | Were transcripts returned to participants for comment and/or correction? | N/A |
| **Domain 3: Analysis and Findings** | | | |

| Topic | Item No. | Guide Questions / Description | Reported on Page No. |
|---|---|---|---|
| Data analysis | | | |
| Number of data coders | **24** | How many data coders coded the data? | 6 |
| Description of the coding tree | **25** | Did authors provide a description of the coding tree? | 6, Table 3 and Supplementary File 3 |
| Derivation of themes | **26** | Were themes identified in advance or derived from the data? | 6; 8-11, Table 3 and Supplementary File 3 |
| Software | **27** | What software, if applicable, was used to manage the data? | N/A |
| Reporting | | | |
| Participant checking | **28** | Did participants provide feedback on the findings? | N/A |
| Quotations presented | **29** | Were participant quotations presented to illustrate the themes/findings? Was each quotation identified? e.g. participant number | 9-11; Supplementary File 3 |
| Data and findings consistent | **30** | Was there consistency between the data presented and the findings? | 8-11; Supplementary File 3 |
| Clarity of major themes | **31** | Were major themes clearly presented in the findings? | 8-11, Table 3 and Supplementary File 3 |
| Clarity of minor themes | **32** | Is there a description of diverse cases or discussion of minor themes? | 9-11 |
