# Supplementary material for: The Intercultural Mediator as a Bridge in Healthcare Professional–Migrant Patient Care Relationships: A Qualitative Study
Source: Healthcare (Basel). 2026 Jun 30;14(13):1903. doi: 10.3390/healthcare14131903 (PMC13360903; doi:10.3390/healthcare14131903)
Supplement: Supplementary file 1 [file healthcare-14-01903-s001.zip › 2_V2.pdf]

2. Semi-Structured Interview Guide

Section	Main question	Essential probe
Opening	Can you tell me about a situation in which you cared for a migrant patient?	How did you experience it?
	What was it like for you to communicate and build a relationship with migrant patients?	Can you describe a specific moment that was particularly significant or challenging?
	What strategies have you used to address communication or relationship barriers?	Which did you find most effective, and why?
	Can you tell me about your experience collaborating with an intercultural mediator?	How did their presence influence your relationship with the patient?
	What meaning does the presence of the mediator have for you in your work?	Were there other professionals who particularly supported you?
	How do you perceive cultural mediation in your hospital setting?	What changes or resources could improve care for migrant patients?
Closing	Is there anything else you would like to share about your experiences caring for migrant patients or working with intercultural mediators?	—
